# The Effect of Green Tea versus Sour Tea on Insulin Resistance, Lipids Profiles and Oxidative Stress in Patients with Type 2 Diabetes Mellitus: A Randomized Clinical Trial

**Published:** 2014-09

**Authors:** Hassan Mozaffari-Khosravi, Zeinab Ahadi, Marziyeh Fallah Tafti

**Affiliations:** 1 Diabetes Research Center of Yazd, Department of Nutrition, Faculty of Health, Shahid Sadoughi University of Medical Sciences, Yazd, Iran;; 2 Health Centre of Taft, Shahid Sadoughi University of Medical Sciences, Yazd, Iran

**Keywords:** Diabetes mellitusGreen tea, Insulin resistance, Oxidative stress

## Abstract

**Background:** By decreasing oxidative stress and whereby decreasing insulin resistance, it may be possible to decrease complications of Diabetes Mellitus (DM). Green tea and sour tea contain phytochemicals which have anti-oxidative function. The aim of this study is to compare the effect of sour and green tea consumption on insulin resistance and oxidative stress in DM.

**Methods:** This study is a randomized clinical trial in which 100 type 2 diabetes patients were randomly assigned into sour tea group (ST) and green tea group (GT). The patients were instructed to drink 150ml sour tea and green tea infusion, respectively, three times a day for 4 weeks. Fasting blood sugar (FBS), fructosamine, lipid profiles, fasting blood insulin (FBI), homeostasis model assessment of insulin resistance (HOMA-IR); beta cell function (b%), insulin sensitivity (S%) and malondialdehyde (MDA) were monitored.

**Results: **HDL-c significantly increased in both groups. The median of FBI in GT showed significant decrease (8.5 to 6.6 μIU/mL) unlike the ST which showed significant increase (8.2 to 16.3 μIU/mL). The median of HOMA-IR after the intervention in GT showed lower levels than the ST (1.1 vs. 1.6, P=0.004). The median of b% only in ST showed significant increase from 38.2% at the baseline to 47.7% after the intervention. The mean of S% only in ST showed significant decrease after the intervention.

**Conclusion: **This study shows that the use of 150 ml infusion of green tea or sour tea, three times a day for four weeks, has positive effect on insulin resistance and certain lipoproteins in type 2 DM.

**Trial Registration Number: **IRCT201107317161N1

## Introduction

Tea is the most common beverage in the world.^1^ There are many kinds of tea such as black, sour, white, green tea etc.^2^ In petals of sour tea flower (Hibiscus Sabdariffa), there is a mixture of different alkaloids like cyanidin 3, rutinocod, delfinidin, inisaldids, galactose, hibcitin, pectin, polysaccharides, mucopolysaccarides, gosptin, ascorbic acid, citric acid, antocyanin, beta-carotene, sitosterol, esteric acid, etc.^3^ Each of these composites can have various effects on the human body.

Different clinical trials regarding the effect of sour tea on humans and animals have shown that this herb has several positive effects like decrease in systolic and diastolic blood pressure.^4^^,^^5^ Phenolic protoctechui acid being present in sour tea, has protective antioxidant effect on the liver.^6^ It also causes apoptosis of cancer cells.^7^ Antocyanin like delphinidin 3-sambubioside present in sour tea has also the same effect.^8^

Green tea (Camellia Sinesis) is enriched with flavonoids, used as herbal medicine worldwide.^9^ This herb is enriched with polyphenols, catechin epicatechin, epigallocatechin and epigallocatechin-3-gallate. It also contains other composites like caffeine, tannins, vitamins, and saponins.^9^^,^^10^ Several different studies indicate that green tea has antioxidant, antimutagenic, antibacterial effect, anti-inflammatory and hypo-cholesterolaemia action.^11^^-^^15^ This is due to the effect of catechin and galic acid present in green tea.^16^ Certain investigations also show that catechin found in green tea inhibits the proliferation of breast cancer cells in vitro studies.^17^

Diabetes mellitus (DM) is the most common and on the rise chronic metabolic disease worldwide.^18^^,^^19^ During 2010, the number of adults with diabetes reached 285 million. It is estimated that by the year 2030, DM would reach up to 439 million.^18^ In Iran, 2 million adults were reported to have diabetes during 2005.^20^ Considering that DM has high morbidity/mortality and due to positive effects observed in several kinds of tea (particularly in sour tea and green tea), investigating the effects of these kinds of tea on DM was imperative.

Clinical trials have shown that in both types of DM, oxidative stress plays an important role in causing the adverse effects of DM. Increase in free oxidized radicals causes increase in peroxidation of lipids and increase in insulin resistance.^21^^,^^22^ Patients with insulin resistance are at risk of developing metabolic syndrome which is the main cause of heart disease, dyslipidaemia and hypertension.^22^ By decreasing oxidative stress and whereby decreasing insulin resistance, it might be possible to decrease incidence and severity of the adverse effects of DM.

As mentioned, green and sour tea both contains flavonoids and various polyphenols which have anti-oxidative and anti-inflammatory function. Due to the limited number of clinical studies on these kinds of tea, this study was instigated to compare the effect of sour tea and green tea on insulin resistance, lipids profiles and oxidative stress in patients with type 2 diabetes.

## Patients and Methods

*Study Design and Participants*

A randomized clinical trial was conducted during 2011-2012 with the participation of 100 patients with type 2 diabetes mellitus under the supervision of Yazd Diabetic Research Centre. 

Inclusion criteria were patients with: (1) age between 30 to 60 years old; (2) at least 5 years DM duration; (3) fasting blood sugar between 80-250mg/dl; (4) no obvious DM complications, like nephropathy etc.; (5) non-insulin dependent; (6) non-smoker or addiction to certain illicit drugs; (7) no history of using dietary supplements like antioxidants, multivitamins with minerals and omega-3 during the last 6 months and (8) no history of organ diseases like renal, thyroid, liver or heart disease. Exclusion criteria were: (1) had a change in their main usual diet; (2) allergy to sour tea or green tea; (3) change in the dose or drug used as glucose-lowering agents and anti-lipid agents and (4) reluctant participation. 

The 100 patients qualified for inclusion were randomly divided into two groups of green tea (GT) and sour tea (ST) users. This was carried out using a sequential list prepared based on tables of random numbers. Both groups were given the specified number of label tea packets having equal weight. The patients under study were advised to prepare the specified tea at home according to the informed instructions on tea preparation, drink tea three times a day (2 h after the main meal) for a period of four weeks.

Before starting the clinical trail, the patients were debriefed on how to prepare the tea bags each containing 3-gram of the specified tea. The participants in GT group were instructed to add one teabag in 150ml of hot water at 60-70^o^C and drink after 5 minutes. The participants in the ST group were instructed to boil the 3-gram sour teabag in 150ml of 60-70^o^C water and wait for 10-15 minutes and then drink the tea. In case of distaste for the tea flavor, consumption of one date palm was allowed.

The patients were requested to follow guidelines: (1) the total quantity of the prepared 150 ml tea should be taken; (2) avoid drinking any other kind of tea during this clinical trial and (3) unaltered daily food diet and physical activity except avoidance of food containing polyphenols like chocolates and coffee. During the intervention, patients were examined regarding allergic reaction or adverse effects due to the specified tea. Each patient was investigated regarding the percent of unused teabags, to check patient’s compliance. To ensure tea quality, the green tea and sour tea were purchased from reliable sources and were approved by naturist practitioners. 

*Measurements*

The demographic data were recorded at the initiation of study and anthropometric and 24-h dietary recall data were measured before and after 4 weeks of the intervention. Weight was measured with minimum clothing using a digital balance with 100g sensitivity. Height was measured without shoes in an erect standing position with a fixed measurement tape with 1mm sensitivity.

Overnight fasting blood samples were obtained before and after intervention. Blood samples were checked for fasting blood sugar (FBS), fructosamine, triglyceride, total cholesterol as well as HDL-c and LDL-c, fasting blood insulin and serum malondialdehyde (MDA). The FBS, triglyceride and total cholesterol were tested by laboratory kit (Pars Azmoon Co Tehran, Iran) by enzyme method glucose-oxidase, glycerol-oxidase and cholesterol oxidase method, respectively. For HDL-c, first- sedimentation of B-lipoprotien by dextran sulfate and magnesium chloride was done. Then, by enzyme method using cholesterol oxidase HDL-c was measured. LDL-c was calculated using Freidwald formula. Fasting blood insulin was measured by ELlSA kits (Padgin Teb Co, Tehran, Iran). Fructosamine was measured by calorimetric method and reduction nitro-blue-tetrazolium. Serum MDA was measured by thiobarbituric acid technique.

Insulin resistance status was assessed using the following indices: Homeostasis model assessment of insulin resistance (HOMA-IR); level of beta cell function (b%) and insulin sensitivity (S%). These were calculated using HOMA calculation software (HOMA calculator, version 2.2.2, Diabetes Trial Unit University of Oxford).

*Ethical Considerations*

Each patient’s information was given a secret code and saved as confidential. This study was proposed and approved by the Ethics-in-Research Commission of Shahid Sadoughi University of Medical Sciences and written informed consent was obtained from each participant. Also it has been registered on the Iranian clinical trial registration (www.irct.ir) as IRCT201107317161N1.

*Statistical Analysis*

Data was analyzed using SPSS version 11 and the dietary intake data were analyzed by Nutritionist IV (Nutritionist IV Diet Analysis, First Data Bank Division, Hearst Corp., San Bruno, CA). The normality of data was checked by Kolmogorov–Smirnov test. The results within normal distribution were expressed as mean±SD otherwise they were reported as percentile of 25, 50 and 75. The paired t-test and Wilcoxon signed ranks test were used to compare variables within a group. Student t-test and Mann-Whitney test were used to compare variables between the groups. Chi-square test was used for comparing qualitative variables between the two groups. P-value less than 0.05 was considered statistically significant.

## Results

**Figure 1:**
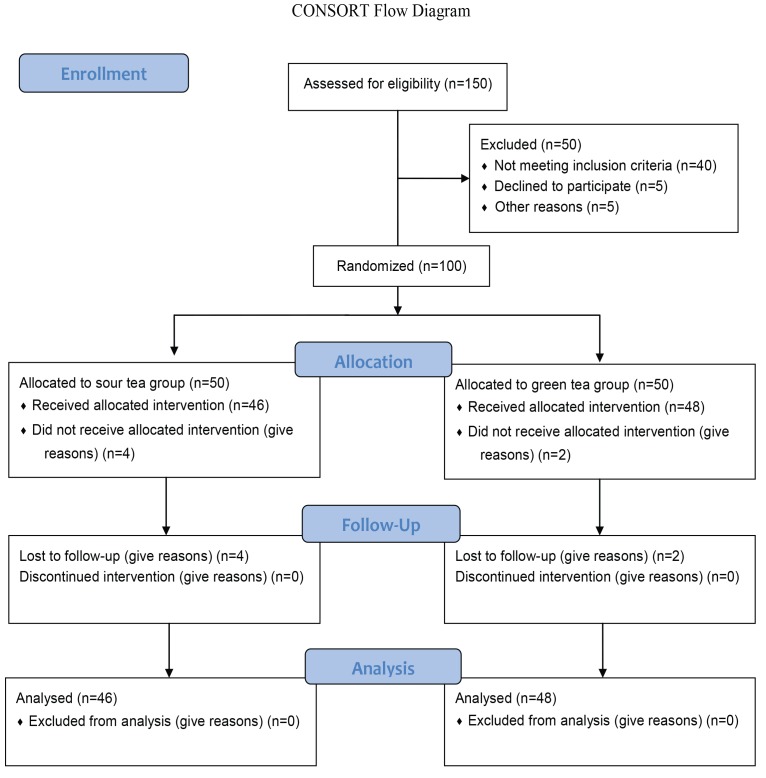
Intervention Steps

Prior to intervention, 100 patients were selected to participate in the clinical trial, out of which 94 remained under investigation till the end of the intervention (figure 1). Among the 6 drop-outs, 4 belonged to the ST and 2 belonged to GT. The reasons for drop-out were allergy to the specified tea, reluctance to continue in the clinical trial, travel and/or being unable to tolerate the taste of the tea. The compliance rate was 89% in the ST and 91% in the GT groups.

Comparison of the qualitative and quantitative variables between the two groups before the intervention is shown in table 1 and table 2. As shown, no significant differences were found for quantitative variables between the groups.

**Table 1:** Comparison of the quantitative variables between the two groups before the intervention

**Variables**	**Sour Tea (46)**	**Green Tea (48)**	P value*
Age (year)	**6±52.1	6.7±52.2	0.9
Age of affliction with diabetes (year)	5.9±48.1	6.6±48.2	0.9
Duration of affliction (year)	25.9±3.6	24.9±3.6	0.8
Weight (kg)	12.6±73.1	12.8±71.8	0.6
Height (cm)	7.9±160.2	10.2±160.6	0.8
Body mass index (kg/m^2^)	3.8±28.3	5.6±28	0.7
Dietary intake			
Energy (Kcal/day)	1555.2±115.9	1503.9±257.3	0.4
Carbohydrate (g/day)	248±49	235±40	0.3
Protein (g/day)	54.4±16.5	50.3±18.3	0.3
Fat (g/day)	38.4±16.6	40.3±12.6	0.5

*Student *t* test; **mean±SD

**Table 2:** Comparison of the qualitative variables between the two groups before the intervention

**Qualitative variables**	**N (%)**	**N (%)**	P value*
Gender			
Male	9 (11.6)	12 (25.0)	0.3
Female	37 (80.4)	36 (75.0)	
Education			
Illiterate	8 (17.4)	10 (20.8)	0.2
Primary	25 (54.3)	18 (37.5)	
High school	7 (15.2)	15 (31.3)	
Higher education	6 (13.0)	5 (10.4)	
Taking oral hypoglycemic agents	42 (91.3)	43 (89.6)	0.50
Taking anti-hypertensive drug	18 (40.0)	20 (42.6)	0.40
Having a special diet	6 (13.0)	10 (20.80)	0.20

*Chi square test

The mean of the study parameters before and after the intervention is shown in table 3. As shown, mean of FBS, fructosamine, triglyceride, total cholesterol, LDL-c, and MDA exhibited no significant differences between the groups as well as within a group. The mean of HDL-c within both groups increased significantly in spite of no differences between the groups before and after the intervention. Mean of insulin sensitivity between the groups, before the intervention, showed no significant differences. However, GT showed significant differences compared with ST after the intervention. The mean of insulin sensitivity before and after intervention within the GT group showed no difference but it showed significant decrease in the ST group. Mean of MDA between the groups showed no differences before and after the intervention.

**Table 3:** Comparing means of study parameters before and after the intervention in sour tea and green tea groups

**Variables**	**Baseline**	**End**	Changes***	P value**
Fasting blood sugar (mg/dl)				
Sour Tea	160.50±49.1	162.10±49.6	-1.6±26	0.60
Green Tea	155.30±47.4	154.00±48.8	1.2±26	0.70
P value*	0.60	0.40	0.50	
Fructosamine (µmol/l)				
Sour Tea	308.20±38.5	309.10±44.4	-0.86±31	0.80
Green Tea	308.50±33.1	307.80±35.5	0.33±21	0.90
P value	0.90	0.80	0.80	
Insulin sensitivity (%)				
Sour Tea	88.10±38.4	70.00±34.1	18.1±31	<0.001
Green Tea	88.60±44.50	96.00±45.3	-7.3±36	0.10
P value	0.90	0.002	<0.001	
Triglyceride (mg/dl)				
Sour Tea	211.6±107.6	216.5±115.7	-4.1±58	0.50
Green Tea	194.9±95.3	192.7±101.8	2.2±58	0.70
P value	0.40	0.20	0.50	
Total cholesterol (mg/dl)				
Sour Tea	190.1±35.7	197.5±38.7	-7.3±30	0.10
Green Tea	194.2±33.0	184.5±36.3	-0.29±22	0.90
P value	0.50	0.70	0.20	
Low density lipoprotein (mg/dl)				
Sour Tea	111.3±21.6	115.8±23.3	-4.5±20	0.10
Green Tea	114.3±22.6	113.0±21.7	1.2±19.5	0.60
P value	0.50	0.50	0.10	
High density lipoprotein (mg/dl)				
Sour Tea	37.1±8.4	41.8±8.8	-4.6±10	0.004
Green Tea	38.7±9.1	43.0±13.0	-4.2±13	0.03
P value	0.0.3	0.6	0.80	
MDA (nmol/ml)				
Sour Tea	1.06±0.4	1.05±0.3	0.01±0.58	0.8
Green Tea	1.10±0.3	1.08±0.3	0.05±0.49	0.4
P value	0.3	0.6	0.70	
Body mass index (kg/m^2^)				
Sour Tea	3.8±28.3	3.8±28.2	0.07±0.3	0.1
Green Tea	5.6±28	5.5±27.9	0.07±0.29	0.08
P value	0.7	0.7		

*Student *t* test; **Paired *t *test; ***‘-‘increase

The median (50^th^ percentile) of some insulin resistance indices before and after the intervention in both groups are shown in table 4. As shown, the median of fasting insulin in GT group showed significant decrease (8.5 to 6.6 μIU/mL) unlike the ST which showed significant increase (8.2 to 16.3 μIU/mL). The median of this variable before the intervention showed no statistically significant difference between the groups. However, after the intervention, the ST group showed a difference being significantly higher than the GT. The median of HOMA-IR index before the intervention showed no significant difference in both groups, but after the intervention the GT group showed lower levels than ST (1.1 vs. 1.6). Also the median of this variable in the ST group, after the intervention, showed significant increase (P=0.002), but in the GT group the median of this showed no significant change (P=0.07). The median of b% before and after the intervention between the groups showed no statistical difference, but only in ST group it showed significant increase from 38.2% at the baseline to 47.7% after the intervention.

**Table 4:** The comparison of insulin resistance indices before and after the intervention in sour tea and green tea groups

**Variables**	**Baseline**			**End**			P value**
	**Percentiles**			**Percentiles**			
	** 25^th^**	** 50^th^**	** 75^th^**	** 25^th^**	** 50^th^**	** 75^th^**	
Fasting blood insulin (μIU/mL)							
Sour tea	6.3	8.2	10.6	7.2	16.3	11	<0.005
Green tea	6.6	8.5	11.2	7.6	6.6	11.9	0.04
P value*		0.70			0.005		
HOMA-IR							
Sour tea	0.9	1.20	1.50	1.0	1.60	2.40	<0.002
Green tea	1.0	1.30	1.70	0.80	1.1	1.75	<0.07
P value		0.60			0.004		
Beta-cell function (%)							
Sour tea	23.90	38.20	52.20	31.60	47.70	70.90	0.023
Green tea	25.20	40.00	58.10	26.10	38.10	66.70	<0.90
P value		0.80			0.20		

Wilcoxon Signed Ranks’ Test;** Mann-Whitney Test

## Discussion

This study shows that drinking the sour tea and green tea infusions, 3 times per day for 4 weeks, has different effects on the insulin resistance and certain lipoprotein in patients with type 2 diabetes mellitus. In both groups of tea users, the HDL-c increased significantly. Insulin sensitivity was seen lesser in sour tea and thus more insulin resistance in sour tea users. Subsequently it appears that both kinds of tea have favorable effect on patients with type 2 diabetes, but green tea has a better effect on insulin resistance indices than the sour tea.

Similar to previous studies,^23^^-^^25^ this study also shows that sour tea has no effect on FBS. Sour tea does not have any significant positive effect on lipoprotein profile except on HDL-c. This was also reported after a clinical trial conducted by Mohagheghi et al.^24^ The same design trial conducted by Mozaffari-Khosravi et al.^23^ shows that sour tea, in addition to having positive effect on HDL-c, has significant effect on decreasing other lipoproteins. Certain studies conclude that the effect of sour tea on lipoprotein is due to water-soluble fibers identified in this tea.^26^

In the same manner as past studies, this study also shows that green tea have no effect on FBS.^27^^-^^31^ However, few others demonstrated that green tea has an effect on FBS.^32^^,^^33^ Few investigators claim that green tea has no effect on lipid profile while Hsu Ch et al.^28^ as well as the present study indicate an increase in HDL-c.

In this clinical trial, as well as others,^34^ it is observed that green tea users have significant decrease in fasting blood insulin. It should be noted that few other clinical trials did not show any change in fasting blood insulin.^35^^,^^36^ The reason behind such discrepancy could be due to the intervention duration and the method of tea brewing. This argument is backed up with the fact that the intervention period in the present study was 4 weeks compared with 8 to 12 weeks in other clinical trials. Furthermore, the green tea used in the past studies was in the form of herbal capsules while in the current trial, the specified tea was prepared in hot water or infusions. At the end of the intervention, mean insulin sensitivity decreased significantly in sour tea users but was less compared with the green tea users (table 3). As much as known, this article is the first attempt in investigating the effect of these kinds of tea on insulin sensitivity as a parameter.

Mean MDA (parameter for oxidative stress) in both tea user groups before and after the intervention shows no difference. In the past human model studies, the effect of sour tea on MDA has not been reported. In two animal model studies, reports shows that sour tea causes decrease of this parameter.^37^^,^^38^ Similar to other studies,^39^^,^^40^ it also shows that green tea had no effect on the mean of MDA parameter. However, by conducting a study on healthy humans, Freese et al.^41^ concludes that green tea causes decrease in MDA. Since these kinds of tea contain several anti-oxidants materials, such conclusion is debatable to various investigators.

In this study, the use of both kinds of tea in patients with type 2 diabetes resulted in significant increase in HDL-c. Also sour tea caused increase in Beta cells function of pancreas and thus an increase in fasting blood insulin. With respect to the current study and other investigations, both kinds of tea have positive effect in patients with dyslipidemia, particularly in patients with diabetes. Accordingly, its consumption is recommended.  

This study had certain weak aspects. For instance, a control group was not included as a third group or the intervention period was too short. In terms of strength, one can mention the comparison of two kinds of tea on a number of parameters such as blood glucose, lipids, insulin resistance and oxidative stress, etc. Note that, up until now, very few such investigations have been carried out. Out of the 100 patients enrolled in this study, 94 patients participated until the end of the intervention. Among these, 90% of patients from both groups successfully followed and executed instructions till the end of the intervention. Besides this, there were no differences regarding qualitative variables and the mean of the quantitative variables between the two groups of specified tea users before the intervention.

It is recommended that other clinical trials should be carried out regarding these herbs and their usefulness i.e. the effect of prolong consumption of these herbs on complications of diabetes mellitus, on insulin dependent diabetic patients and on gestational diabetic patients.

## Conclusion

This study shows that daily use of 150 ml infusion of green tea or sour tea, three times a day for four weeks, has positive effect on insulin resistance and certain lipoproteins in patients with type 2 DM. Using these kinds of tea, particularly the green tea, is recommended in patients with type 2 DM.
